# Upregulation of CD109 Promotes the Epithelial-to-Mesenchymal Transition and Stemness Properties of Lung Adenocarcinomas via Activation of the Hippo-YAP Signaling

**DOI:** 10.3390/cells10010028

**Published:** 2020-12-25

**Authors:** Kang-Yun Lee, Tai-Chih Kuo, Chih-Ming Chou, Wen-Jing Hsu, Wei-Cheng Lee, Jia-Zih Dai, Sheng-Ming Wu, Cheng-Wei Lin

**Affiliations:** 1 Division of Pulmonary Medicine, Department of Internal Medicine, Shuang Ho Hospital, Taipei Medical University, New Taipei 23561, Taiwan; leekangyun@tmu.edu.tw (K.-Y.L.); chitosan@tmu.edu.tw (S.-M.W.); 2 Division of Pulmonary Medicine, Department of Internal Medicine, School of Medicine, College of Medicine, Taipei Medical University, Taipei 11031, Taiwan; 3 Department of Biochemistry and Molecular Cell Biology, School of Medicine, College of Medicine, Taipei Medical University, 250 Wu-Xing Street, Taipei 11031, Taiwan; tckuo@tmu.edu.tw (T.-C.K.); cmchou@tmu.edu.tw (C.-M.C.); m120107025@tmu.edu.tw (W.-J.H.); m120107021@tmu.edu.tw (W.-C.L.); jasonqaz1118@gmail.com (J.-Z.D.); 4 Graduate Institute of Medical Sciences, College of Medicine, Taipei Medical University, Taipei 11031, Taiwan; 5 Center for Cell Therapy and Regeneration Medicine, Taipei Medical University, Taipei 11031, Taiwan; 6 Cell Physiology and Molecular Image Research Center, Wan Fang Hospital, Taipei Medical University, Taipei 11031, Taiwan; 7 Drug Development and Value Creation Research Center, Kaohsiung Medical University, Kaohsiung 807, Taiwan

**Keywords:** CD109, EMT, lung adenocarcinoma, stemness, YAP

## Abstract

Metastasis is the leading cause of death in lung adenocarcinomas. Identifying potential prognostic biomarkers and exploiting regulatory mechanisms could improve the diagnosis and treatment of lung cancer patients. We previously found that cluster of differentiation 109 (CD109) was upregulated in lung tumor tissues, and CD109 overexpression was correlated with the invasive and metastatic capacities of lung adenocarcinoma cells. However, the contribution of CD109 to lung tumorigenesis remains to be elucidated. In the present study, we identified that CD109 was upregulated in metastatic lung adenocarcinoma cells, and elevation of CD109 was correlated with epithelial-to-mesenchymal transition (EMT) traits in patients with lung adenocarcinoma. Functionally, CD109 expression was crucial for EMT gene expressions, tumor invasiveness, and cancer stemness properties. Moreover, elevation of CD109 was accompanied by upregulation of the yes-associated protein (YAP) signature in metastatic lung cancer cells and lung cancer patients, and activation of YAP was demonstrated to participate in CD109-elicited EMT gene expressions and tumor invasiveness. Our study reveals the molecular mechanism underlying CD109 in lung tumor aggressiveness, and CD109 could be a potential diagnostic and therapeutic target for lung cancer patients.

## 1. Introduction

Lung cancer is the leading cause of cancer-associated deaths worldwide, and its incidence continues to increase every year. Lung cancer is classified into two major categories of small-cell lung cancer (SCLC) and non-SCLC (NSCLC). NSCLC accounts for 85% of all lung carcinomas and is comprised of squamous carcinomas, large-cell carcinomas, and adenocarcinomas [1]. Although chemotherapy and radiation therapy show responses during the early treatment of NSCLC, molecular changes in NSCLC are a major problem which lead to resistance and distant metastasis [2,3]. Therefore, finding new diagnostic markers and elucidating the underlying mechanisms are urgent needs for lung cancer therapy.

Cluster of differentiation 109 (CD109) is a glycosylphosphatidylinositol (GPI)-anchored protein which belongs to the α2-macroglobulin/C3, C4, and C5 family of thioester-containing proteins. CD109 was found to be expressed by a subset of hematopoietic cells including platelets, endothelial cells, bone marrow CD34+ cells, and activated T cells [4]. It is rarely expressed in normal human tissues but was found to be upregulated in a variety of malignancies including squamous cell carcinomas [5], glioblastomas [6], melanomas [7], pancreatic ductal adenocarcinomas, and lung and breast cancers [8]. Platelet CD109 carries the biallelic platelet-specific alloantigen, Govα/β, which plays a major role in neonatal alloimmune thrombocytopenia [9]. In tumor cells, CD109 expression was reported to regulate epidermal growth factor receptor (EGFR) and Janus kinase (JAK)/signal transducer and activator of transcription (STAT) signaling pathways [10,11], and CD109 decreased sensitivity to EGFR-tyrosine kinase inhibitor (TKI) therapy in lung adenocarcinoma cells [12]. In cervical squamous cell carcinoma, CD109 expression enhanced aggressiveness and maintained cancer stemness properties through the EGFR-STAT3 signaling cascade [13], suggesting the potential role of CD109 as a diagnostic cancer marker and therapeutic target. However, as a co-receptor of the transforming growth factor (TGF)-β receptor (TGF-βR) [14,15,16], CD109 was reported to negatively regulate the epithelial-to-mesenchymal transition (EMT) by antagonizing TGF-β signaling in squamous cell carcinomas [17]. Thus, clarifying the molecular functions and underlying regulatory mechanisms of CD109 in tumorigenesis is urgently needed.

Our recent study found that CD109 expression was associated with aggressiveness and metastasis of lung adenocarcinomas [12]. To further validate the molecular mechanism underlying CD109-mediated tumor aggressiveness, a metastatic CL-LM subline from parental CL1-5 lung adenocarcinoma cells was established in the present study. We identified that CD109 was elevated in metastatic CL-LM cells, and this was correlated with EMT characteristics. Furthermore, expression of CD109 regulated yes-associated protein (YAP) signaling, thereby promoting the EMT, stem cell gene expressions, and cancer stemness properties.

## 2. Results

### 2.1. CD109 Is Upregulated in Metastatic Lung Adenocarcinoma Cells and Is Associated with EMT Gene Expressions

**Figure 1:**
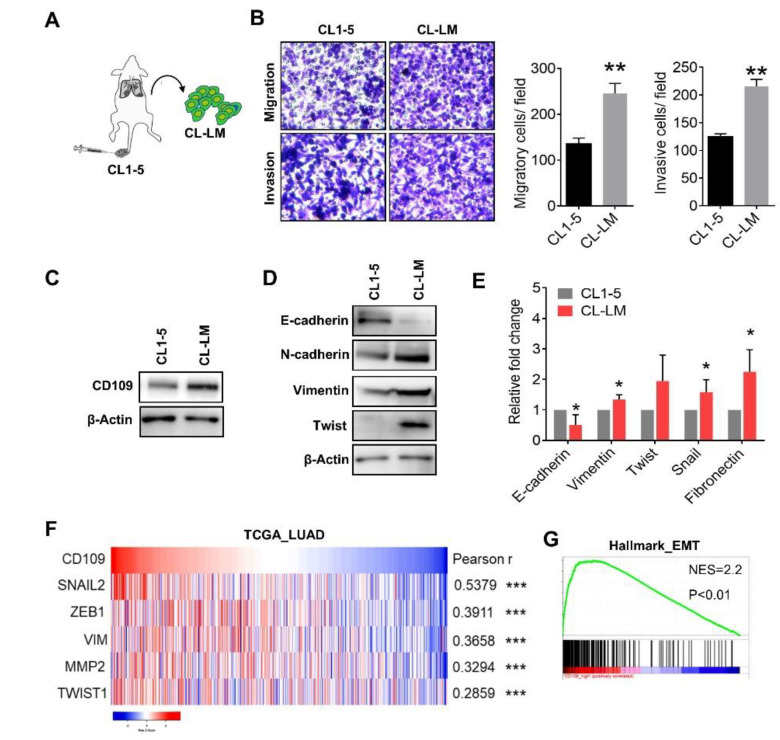
Elevation of CD109 is associated with the epithelial-to-mesenchymal transition (EMT) in metastatic lung adenocarcinoma cells. (**A**) Illustration of the generation of lung metastatic CL-LM cells. Parental CL1-5 lung adenocarcinoma cells were intravenously injected into the tail vein of NOD/SCID mice for 60 days. Metastatic CL-LM cells were isolated from tumor nodules of mice lungs. The detailed methodology is described in “Materials and Methods”. (**B**) Comparisons of the capabilities of migration and invasion between parental CL1-5 and metastatic CL-LM cells. Tumor migration and invasion were evaluated by a transwell analysis. Pictures were taken at 200× magnification. Data are the mean ± SD. * *p* < 0.05; ** *p* < 0.01, as determined by an unpaired *t*-test. (**C**,**D**) Western blot analysis of CD109 and EMT protein markers in CL1-5 and CL-LM cells. (**E**) Real-time PCR analysis of EMT gene expressions in CL1-5 and CL-LM cells. (**F**) Heatmap plot of transcriptome levels of CD109 and EMT-related genes in TCGA lung adenocarcinoma patients (TCGA_LUAD). Associations of transcriptome levels between CD109 and EMT-related genes were analyzed by Pearson correlations. * *p* < 0.05; ** *p* < 0.01; *** *p* < 0.001. (**G**) Enrichment plot between CD109 and the EMT signature from a Hallmark gene set in lung cancer patients using GSEA. NES, net enrichment score.

We previously identified that CD109 is upregulated in lung adenocarcinoma cells, and suppression of CD109 inhibits the metastatic capacity [12]. To further investigate associations of CD109 with lung cancer metastasis, lung adenocarcinoma CL1-5 cells were intravenously injected into immunocompromised mice, and CL-LM subline cells from metastatic lung tumor nodules were isolated (Figure 1A). Transwell analyses confirmed upregulation of the migratory and invasive capacities of metastatic CL-LM cells, compared to parental CL1-5 cells (Figure 1B). Western blot analysis showed that the CD109 protein level increased in metastatic CL-LM cells (Figure 1C), and results of the Western blot and real-time PCR assays showed that expressions of mesenchymal-related genes were concomitantly upregulated in CL-LM cells (Figure 1D,E). Analysis of TCGA database showed that the CD109 transcriptional level was positively correlated with EMT-related genes, including SNAI2, ZEB1, VIM, MMP2, and TWIST1, in patients with lung adenocarcinomas (Figure 1F), and Gene Set Enrichment Analysis (GSEA) revealed that high CD109 expression in lung cancer patients was associated with the EMT signature (Figure 1G). These data suggest that CD109 may regulate EMT properties and tumor mobility.

### 2.2. Expression of CD109 Regulates the EMT and Tumor Invasiveness in Lung Adenocarcinoma Cells

**Figure 2:**
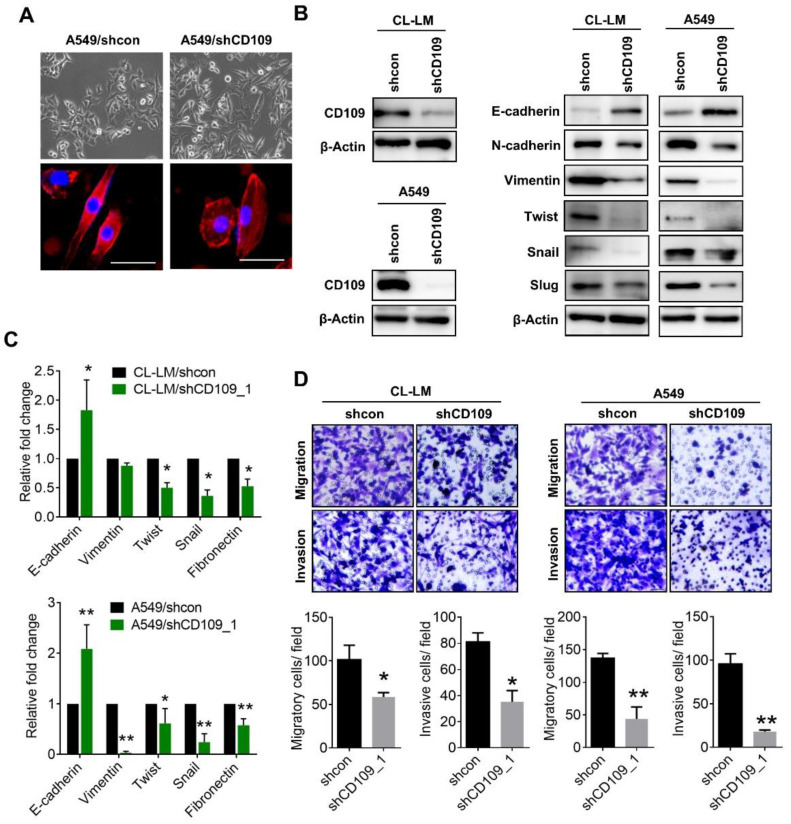
Suppression of CD109 downregulates epithelial-to-mesenchymal transition (EMT) gene expressions and mobility in lung adenocarcinoma cells. (**A**) Bright field images of cell morphology in A549/sh-control and A549/shCD109 cells (upper panel). Fluorescent images of actin filaments in A549/sh-control and A549/shCD109 cells (lower panel). Scale bar = 50 μm. (**B**) Western blot analyses of CD109 (left panel) and EMT-related protein levels (right panel) in CD109-knockdown A549 and CL-LM cells. (**C**) CD109-knockdown suppressed EMT-related gene expressions, as determined by real-time PCR assay. (**D**) Transwell analyses of migratory and invasive capacities of CD109-knockdown A549 and CL-LM cells. Data were derived from three random fields by three independent experiments, and results are expressed as the mean ± SD. * *p* < 0.05; ** *p* < 0.01, as determined by an unpaired *t*-test.

**Figure 3:**
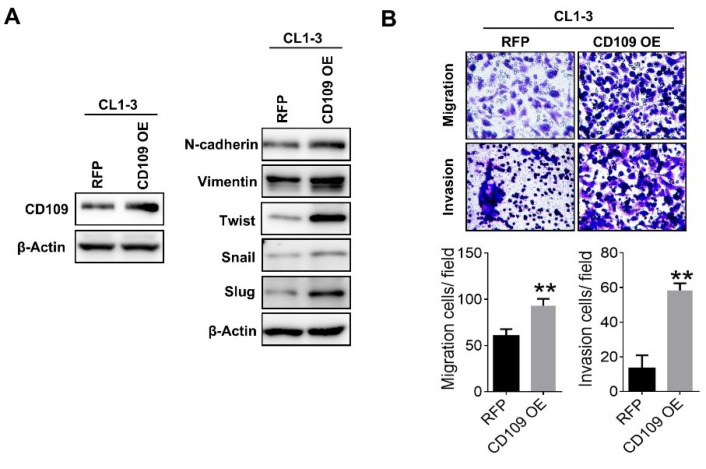
Overexpression of CD109 promotes the epithelial-to-mesenchymal transition (EMT) and tumor mobility. (**A**) Western blot analyses of CD109 (left panel) and EMT-related protein levels (right panel) in CD109-overexpressing CL1-3 cells. RFP, red fluorescent protein. (**B**) CL1-3 cells were transiently transfected with the CD109 plasmid, and migratory and invasive capacities were measured by transwell analyses. Data were derived from three random fields by three independent experiments, and results are expressed as the mean ± SD. ** *p* < 0.01, as determined by an unpaired *t*-test.

To validate that CD109 participates in EMT regulation, we knocked-down CD109 in A549 cells, which were identified as overexpressing CD109 [12]. Inhibition of CD109 in A549 cells exhibited a flat and polygonal morphology, compared to the control knockdown A549 cells which showed an elongated shape (Figure 2A). F-actin staining also showed that knockdown of CD109 decreased actin filament polarity, which is a feature of cellular mobility (Figure 2A). Moreover, CD109-knockdown in A549 cells downregulated EMT-associated transcriptional factors including Snail, Slug, and Twist, and this was accompanied by reductions in mesenchymal markers, such as vimentin and N-cadherin. On the contrary, the epithelial marker, E-cadherin, increased in CD109-knockdown A549 cells (Figure 2B). Similarly, the real-time PCR analysis showed that expressions of EMT-associated genes were modulated by CD109 (Figure 2C). Furthermore, suppression of CD109 was confirmed to decrease migration and invasion of A549 cells (Figure 2D). Consistent results were observed in CL-LM cells in which inhibition of CD109 attenuated EMT-related gene expressions and tumor mobility (Figure 2B–D). To validate that CD109 promotes the EMT and invasiveness of lung tumor cells, CD109 was ectopically overexpressed in low-invasive CL1-3 cells. Results showed that CD109 overexpression increased mesenchymal markers (Figure 3A). Moreover, CD109 overexpression significantly promoted the migratory and invasive capacities of CL1-3 cells (Figure 3B), confirming that CD109 expression is responsible for EMT traits in lung adenocarcinoma cells.

### 2.3. CD109 Expression Regulates Stemness Properties

**Figure 4:**
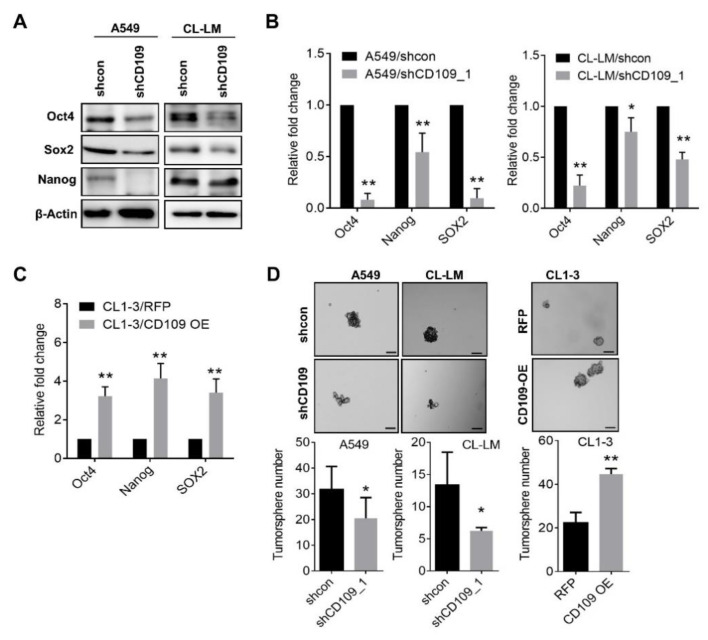
Expression of CD109 is associated with stemness properties. (**A**) Real-time PCR analysis of stem-like gene expressions in control and CD109-silenced A549 and CL-LM cells. (**B**) Western blot analysis of stem-like protein levels in control and CD109-silenced A549 and CL-LM cells. (**C**) Real-time PCR analysis of stem-like gene expressions in control and CD109-overexpressed CL1-3 cells. (**D**) Representative images of tumorsphere formation in CD109-silenced A549 and CL-LM cells (left panel) and CD109-overexpressing CL1-3 cells (right panel). Data were derived from three independent experiments, and results are expressed as the mean ± SD. * *p* < 0.05; ** *p* < 0.01, as determined by an unpaired *t*-test. Scale bar, 50 μm.

It is well documented that upregulation of EMT traits increases cancer stemness activity. Thus, we further evaluated the involvement of CD109 in stemness properties in lung cancer cells. Results of the real-time PCR and Western blot analyses showed that mRNA and protein levels of stemness markers, including Oct4, Nanog, and Sox2, were significantly downregulated in CD109-knockdown A549 and CL-LM cells (Figure 4A,B). Conversely, overexpression of CD109 increased expression of stemness genes in CL1-3 cells (Figure 4C). Moreover, the formation of tumorspheres, which refers to the self-renewal ability of stem-like tumor cells, significantly decreased in CD109-knockdown A549 and CL-LM cells (Figure 4D). Conversely, CD109 overexpression increased tumorsphere formation in CL1-3 cells (Figure 4D), indicating that CD109 expression is crucial for stem-like properties in lung adenocarcinoma cells.

### 2.4. Activation of YAP Participates in the CD109-Mediated EMT and Invasiveness

**Figure 5:**
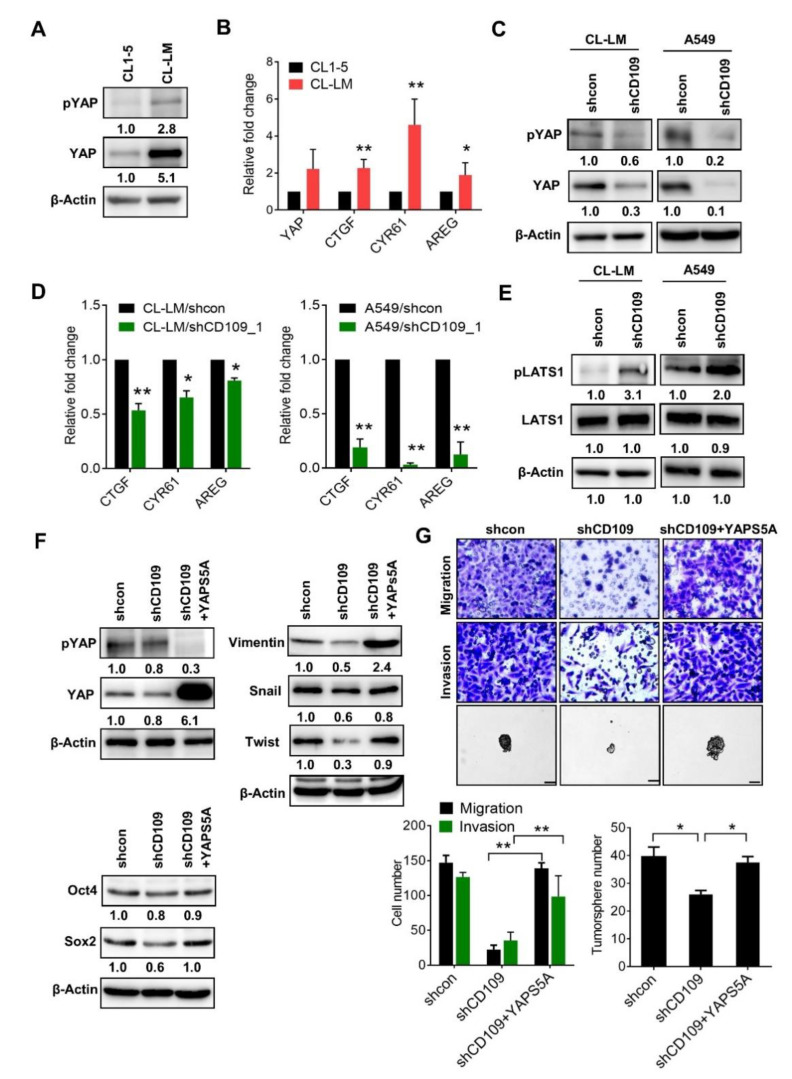
Expression of CD109 regulates YAP. (**A**) Western blot analysis of YAP and phosphorylated YAP protein levels in CL1-5 and CL-LM cells. Quantitative analyses were performed using Image J software. (**B**) Real-time PCR analysis of YAP and YAP downstream gene expressions in CL1-5 and CL-LM cells. (**C**) Western blot analysis of YAP and phosphorylated YAP protein levels in CD109-silenced A549 and CL-LM cells. (**D**) Real-time PCR analysis of YAP downstream gene expressions in CD109-silenced A549 and CL-LM cells. (**E**) Western blot analysis of phosphorylated LATS1 protein level in CD109-silenced A549 and CL-LM cells. (**F**) Western blot analysis of EMT- and stemness-related protein levels in A549/sh-control, shCD109, and shCD109+YAPS5A cells. (**G**) Representative images of tumor migration, invasion, and tumorsphere formation in A549/sh-control, shCD109, and shCD109+YAPS5A cells. Data were derived from three random fields by three independent experiments, and results are expressed as the mean ± SD. Quantification of protein bands from Western blot was carried out by Image J software. * *p* < 0.05; ** *p* < 0.01, as determined by an unpaired *t*-test. Scale bar, 50 μm.

We previously identified that YAP, transducer of the Hippo pathway, is increased in metastatic tumor cells [18], and elevation of YAP/TAZ participates in EGFR-TKI sensitivity in lung cancer cells [19]. To further elucidate the molecular mechanisms of CD109-mediated tumor aggressiveness, we examined the involvement of YAP signaling. As shown in Figure 5A, Western blot analysis revealed that the YAP protein level drastically increased in metastatic CL-LM cells, compared to CL1-5 cells (Figure 5A). The ratio of phosphorylated YAP to total YAP decreased in CL-LM cells (Figure 5A), indicating that activated YAP was upregulated in metastatic lung tumor cells. We further examined mRNA levels of YAP and YAP downstream by real-time PCR assay. Results showed that YAP mRNA had slightly but insignificantly increased in CL-LM cells (Figure 5B). However, YAP downstream targets, including CTGF, CYR61, and AREG, were significantly upregulated in CL-LM cells (Figure 5B). To validate whether CD109 regulates YAP signaling, YAP and p-YAP levels were measured in CD109-knockdown A549 and CL-LM cells. As shown in Figure 5C,D, CD109 suppression resulted in reductions in total and phosphorylated YAP in A549 and CL-LM cells (Figure 5C), and the expression of YAP downstream targets, including CTGF, CYR61, and AREG, were downregulated in CD109-knockdown cells (Figure 5D). Moreover, we found that CD109 suppression increased the phosphorylation of LATS in A549 and CL-LM cells (Figure 5E), suggesting that CD109 activates YAP via a canonical Hippo signaling event. To further clarify that activation of YAP participates in CD109-regulated tumor invasiveness and stemness, a constitutively activated YAP plasmid (YAPS5A) was transfected into CD109-knockdown A549 cells. Results showed that enforced expression of YAPS5A restored EMT- and stemness-related protein levels in A549/shCD109 cells (Figure 5F). Likewise, CD109 suppression resulted in the downregulations of tumor invasiveness and tumorsphere formation were restored by the ectopic expression of YAP (Figure 5G). These data validate that YAP activation participates downstream of CD109 in promoting lung tumor aggressiveness.

### 2.5. Clinical Association of CD109 and the YAP Signature

**Figure 6:**
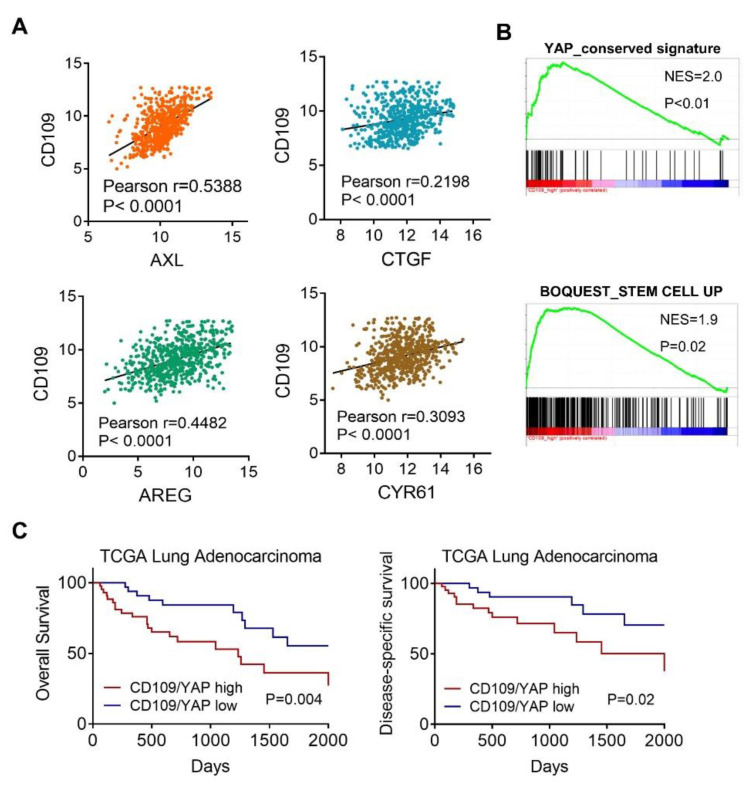
Expression of CD109 is associated with the YAP signature in lung adenocarcinoma patients. (**A**) Associations of CD109 and YAP downstream genes in TCGA lung adenocarcinoma patients (LUAD). Correlations were determined using Pearson’s test. (**B**) GSEA plots of enrichment of YAP and stem cell signatures with CD109 in lung cancer patients. (**C**) Disease-specific survival (DFS) and overall survival (OS) probabilities in lung adenocarcinoma patients stratified by CD109/YAP_high and CD109/YAP_low groups. The survival probability was analyzed by a log-rank (Mantel-Cox) statistical test.

We further examined the clinical association between CD109 and YAP signaling from TCGA lung adenocarcinoma dataset (LUAD), and results showed that CD109 expression was positively correlated with YAP downstream targets including AXL (Pearson r = 0.539), AREG (Pearson r = 0.448), CTGF (Pearson r = 0.219), and CYR61 (Pearson r = 0.309) (Figure 6A). Additionally, the GSEA revealed significant associations between CD109 and YAP signatures in lung cancer patients (Figure 6B), and positive correlations of CD109 and stem-like signatures (Figure 6B), confirming the in vitro findings that CD109 regulates YAP signaling. Moreover, lung adenocarcinoma patients with higher coexpression of CD109 and YAP showed worse overall and disease-specific survival probabilities (Figure 6C), underpinning the potential role of CD109 possibly serving as prognostic and therapeutic targets for lung adenocarcinoma patients.

## 3. Discussion

Despite several studies having reported CD109 upregulation in a variety of malignant tumors, its molecular function and detailed regulatory mechanism are still largely unknown. Although the literature reports that CD109 is highly expressed in squamous cell carcinoma [13,20], our previous study identified that CD109 overexpression was specifically associated with poor survival probability in patients with lung adenocarcinoma but not with squamous cell carcinoma [12]. In the current study, we further identified that CD109 expression was significantly associated with EMT and stem-like signatures, and activation of YAP participated in the CD109-elicited EMT and cancer stemness traits. In lung adenocarcinoma patients, coexpression of CD109 and YAP rendered a worse survival prognosis.

CD109 is a glycosyl phosphatidylinositol (GPI)-anchored protein and was reported to be cleaved by furinase [15]. Soluble CD109 is associated with the TGF-βR and results in negative regulation of TGF-β-mediated cellular events. A recent study reported that CD109 expression blocked the TGF-β-elicited EMT process [17]; however, CD109 overexpression was shown to alleviate TGF-β-mediated suppression of cell growth in oral squamous cell carcinoma [5]. Additionally, CD109 was elevated in aldehyde dehydrogenase 1 (ALDH1)-positive epithelioid sarcoma cells, and CD109 expression increased ALDH1 activity by suppressing the TGF-β/Smad signaling pathway [21]. ALDH1 participates in drug detoxification and is well recognized as a surface marker for cancer stem cells [22], implying that CD109 expression might associate with stem-like feature. The Janus face of TGF-β’s effect on different tumor development stages indicates that other intracellular mechanisms may crosstalk with CD109 in addition to its association with TGF-β signaling. Previous studies reported that CD109 is associated with and regulates the EGFR [11,12]. In cervical squamous cell carcinoma, CD109 expression enhanced EGFR-induced phosphorylation of STAT3, resulting in increased tumor aggressiveness and stemness activity [13]. We previously reported that CD109 is responsible for EGFR-TKI sensitivity in lung tumor cells through regulating the EGFR-AKT-mammalian target of rapamycin (mTOR) signaling cascade [12]. Additionally, CD109 acts as a metastatic driver in lung cancer via regulating JAK/STAT3 [10]. Of note, a recent study showed that CD109 expression in glioma stem cells was not associated with TGF-β or STAT3 signaling pathways, but instead regulates YAP/TAZ [23]. Our data were in line with previous findings that CD109 promotes YAP signaling in lung adenocarcinoma cells. Moreover, activation of YAP participated in CD109-mediated EMT and cancer stemness properties. We found that CD109 and YAP were concomitantly upregulated in metastatic CL-LM cells, and expression of CD109 is crucial for YAP downstream gene expressions. Enforced expression of YAP rescued EMT gene expressions, and tumor migration and invasion in CD109-silenced cells. Clinically, CD109 expression is associated with the YAP signature, and coexpression of CD109/YAP renders the worst survival outcome in lung cancer patients. Contrarily, a recent study argued that CD109 suppression did not affect YAP downstream gene expressions in pancreatic ductal adenocarcinoma cells [24]. These findings imply that CD109 may regulate different intracellular mechanisms in a context-dependent manner. Thus, more evidence from future studies is needed to clarify signaling event crosstalk with CD109.

YAP and TAZ are transducers of the Hippo pathway, and dysregulation of YAP/TAZ has been well documented to play crucial roles in tumor growth, stemness, metastasis, and drug resistance [25,26]. Activation of YAP/TAZ can be regulated by the canonical Hippo signaling cascade or by a non-canonical intracellular signaling mechanism such as actin dynamic, G protein couple receptor (GPCR), or β-catenin [27,28]. Minata et al. identified that CD109-positive glioma stem cells are highly enriched in tumor-initiating and radiation-resistant properties [23]. They also found that suppression of CD109 downregulated the YAP/TAZ signature and decreased the TAZ protein level in glioma stem cells. Likewise, Gangwani et al. reported that the Drosophila Tep1 gene (ortholog of human CD109) regulates Yki (the Drosophila ortholog of human YAP/TAZ) using a Drosophila glioma cell model [29]. However, the molecular mechanism through which CD109 regulates YAP/TAZ was not clarified in the abovementioned studies. Our previous study has also identified that CD109 regulates EGFR-AKT-mTOR signaling cascade, and knockdown of CD109 in A549 cells significantly suppressed lung metastasis in vivo [12]. Our present study further discovered that CD109 regulates EMT and stemness of lung adenocarcinoma cells via a YAP-dependent manner. Moreover, CD109 inhibition induced phosphorylation of LATS, and subsequently increased YAP phosphorylation, suggesting that CD109 may regulate YAP by a Hippo signaling cascade. EMT is an evolutionarily conserved process that occurs during development and is important for tumor progression. Although CD109 is reported that negatively regulates TGFβ signaling, which is an inducer of EMT. Paradoxical roles of TGFβ signaling can be either promote or suppress tumor formation, suggesting that CD109 regulates EMT in a cellular context manner and that also highlights the crucial role of CD109-YAP/TAZ signaling pathway in regulating EMT and cancer stemness. Interestingly, elevation of YAP/TAZ was reported to participate in resistance to EGFR-TKI therapies by lung cancer cells [19,30,31,32]. Our previous study also identified that CD109 expression contributes to sensitivity to EGFR-TKI by crosstalk with EGFR signaling [12]. This evidence implies the important role of CD109 in EGFR-TKI therapies, and it also highlights translational relevance of CD109 as a diagnosis and therapeutic target in lung adenocarcinoma patients. Targeting CD109 could provide therapeutic benefits against lung cancer metastasis and drug resistance.

## 4. Materials and Methods

### 4.1. Cell Lines and Reagents

The human A549 lung adenocarcinoma cell line was purchased from the Bioresource Collection Research Center (BCRC, Hsinchu, Taiwan). CL1-5 and CL1-3 cells which respectively exhibit high-invasive and low-invasive capabilities, were provided by Prof. Hsiao-Chi Chung (Taipei Medical University). CL-LM metastatic subline cells were established by intravenously injecting CL1-5 cells into the tail vein of nonobese diabetic/severe combined immunodeficiency (NOD/SCID) mice for 60 days. Tumor-bearing mice were sacrificed, and mice lungs were excised and minced into small pieces. Lung tissues were then incubated with 0.25% trypsin and type IV collagenase (Sigma, St. Louis, MO, USA) for 2 h at 37 °C. Disassociated tumor cells that had migrated out of the lung tissues were washed with phosphate-buffered saline (PBS) and expanded by continuous cultivation for at least 2 weeks [18]. The CL-LM cells are a mixed clone from three mice lung tissues. Cells were maintained in RPMI medium supplemented with 7% fetal bovine serum (FBS), 1% antibiotic-antimycotic, and 1% GlutaMAX (Thermo Fisher Scientific, New York, USA). An antibody against CD109 was purchased from Santa Cruz Biotechnology (Santa Cruz, CA, USA). Antibodies against YAP, LATS, phosphorylated (p)-LATS, and p-YAP were purchased from Cell Signaling Technologies (Beverly, MA, USA). Antibodies for E-cadherin, vimentin, Snail, Slug, Twist, Oct4, Nanog, Sox2, and β-actin were obtained from GeneTex (San Antonio, TX, USA). The CD109 open reading frame (ORF) in pCMV3 was purchased from Sino Biological (Wu-Han, China). The pCMV-flag-YAPS5A plasmid was kindly provided by Addgene (#27371).

### 4.2. Short Hairpin RNA and Lentiviral Infection

shRNAs for human CD109 (TRCN0000073649) were obtained from the National RNAi Core Facility (Academia Sinica, Taipei, Taiwan). The shRNA target sequence is GCCGATCCTTACATAGATATT. Lentiviral preparation and viral infection were performed as previously described [19]. In brief, HEK293T cells were cotransfected with pLKO. shRNA together with the pCMV-∆R8.91 and pMDG plasmids. At 48 h post-transfection, virus-containing supernatants were mixed with polybrene (8 μg/mL) and incubated with target cells for another 48 h. Transduced cells were selected using puromycin (5 μg/mL). A control shRNA targeting the red fluorescent protein (RFP) was used as a negative control.

### 4.3. Transwell Assay

Cells at 1–2 × 10^5^ cells/well suspended in serum-free medium were seeded in 24-well transwell inserts (8-µm pore size, Corning Costar, New York, NY, USA) coated with Matrigel (BD Biosciences, Franklin Lakes, NJ, USA) or left uncoated for 24 or 48 h. Serum-containing medium was added to the bottom wells of the transwell as a chemoattractant. Cells in the upper wells were removed with a cotton swab, and cells which had migrated to the lower well were fixed with methanol, stained with crystal violet solution, and observed under a microscope with a 20× objective (total magnification 200×). The number of cells in three randomly selected fields was counted.

### 4.4. Western Blotting

Cells were lysed in ice-cold radioimmunoprecipitation assay (RIPA) buffer supplemented with a protease and phosphatase inhibitor cocktail (Millipore, Bedford, MA, USA). Equal amounts of proteins were separated by sodium dodecylsulfate (SDS)-polyacrylamide gel electrophoresis (PAGE) and transferred to 0.45-µm-pore-size polyvinylidene difluoride (PVDF) membranes. Membranes were blocked with 1% bovine serum albumin/TBST blocking buffer at room temperature for 30 min and then incubated overnight at 4 °C with specific primary antibodies. Membranes were washed with TBST wash buffer followed by incubation with a horseradish peroxidase-conjugated secondary antibody at room temperature for 1 h. Bands were detected with an enhanced chemiluminescence (ECL) system (Millipore, Bedford, MA, USA). Western blotting was performed at least three times, and representative experiments are shown. Quantification of protein bands from Western blot was carried out by Image J software.

### 4.5. Tumorsphere Formation Assay

Lung adenocarcinoma cells (10^3^ cells) were seeded in six-well ultra-low-attachment plates (Corning Costar). Cells were maintained in serum-free Dulbecco’s modified Eagle medium (DMEM)-F12 supplemented with 20 ng/mL of epidermal growth factor (EGF; PeproTech, Rocky Hill, NJ, USA), 25 ng/mL basic fibroblast growth factor (bFGF; PeproTech), and B27 (Gibco) for 10 days. Formation of tumorspheres was observed and counted under a light microscope.

### 4.6. Real-Time Polymerase Chain Reaction

Target cell total RNA was extracted with a GENEzolTM TriRNA Pure kit (Geneaid Biotech, Taipei, Taiwan) and reverse-transcribed with a TOOLS Easy Fast RT kit (Biotools, Taipei, Taiwan). Complementary DNA was amplified with EvaGreen Master Mix (Biotium, Hayward, CA, USA) in a StepOne Plus Real-Time PCR system (Applied Biosystems, Darmstadt, Germany) with specific primers as previously described [12,18,33]. Results were calculated using the ΔΔCT equation and are expressed as multiples of change relative to a control sample [33].

### 4.7. Bioinformatic Analyses

Gene expression patterns of CD109, EMT- and YAP-associated genes, and overall and disease-specific survival prognostic values from The Cancer Genome Atlas (TCGA) lung adenocarcinoma (LUAD) dataset were downloaded from the University of California, Santa Cruz (UCSC) Xena browser (https://xenabrowser.net/). A high-expression group was defined as occurring in greater than 30% of patients. Associations of CD109 with hallmark_EMT and YAP_conserved signatures in the GSE31210 cohort [34,35,36] were analyzed using a gene set enrichment analysis (GSEA) algorithm.

### 4.8. Statistical Analyses

Data are presented as the mean ± standard deviation (SD) of three independent experiments. Statistical significance was determined by an unpaired, two-tailed Student’s *t*-test unless stated otherwise. * *p* < 0.05; ** *p* < 0.01. A correlation coefficient was analyzed by the Pearson test. The survival probability was plotted by Kaplan-Meier and analyzed by a log-rank (Mantel-Cox) statistical test. All statistical analyses were carried out with GraphPad Prism 6.0 software (San Diego, CA, USA).

## 5. Conclusions

In summary, we demonstrate that expression of CD109 regulates YAP signaling, thereby promoting the EMT, stem cell gene expressions, and cancer stemness properties. CD109 could be a potential diagnostic and therapeutic target for lung cancer patients. Targeting CD109 could provide therapeutic benefits against lung cancer metastasis and drug resistance.
